# Growth Inhibitory Signaling of the Raf/MEK/ERK Pathway

**DOI:** 10.3390/ijms21155436

**Published:** 2020-07-30

**Authors:** Pui-Kei Wu, Andrew Becker, Jong-In Park

**Affiliations:** 1 Department of Biochemistry, Medical College of Wisconsin, Milwaukee, WI 53226, USA; abecker@mcw.edu; 2 Department of Surgery, Medical College of Wisconsin, Milwaukee, WI 53226, USA

**Keywords:** Raf, MEK1/2, extracellular signal-regulated kinase 1 and 2 (ERK1/2), growth arrest, cell death

## Abstract

In response to extracellular stimuli, the Raf/MEK/extracellular signal-regulated kinase (ERK) pathway regulates diverse cellular processes. While mainly known as a mitogenic signaling pathway, the Raf/MEK/ERK pathway can mediate not only cell proliferation and survival but also cell cycle arrest and death in different cell types. Growing evidence suggests that the cell fate toward these paradoxical physiological outputs may be determined not only at downstream effector levels but also at the pathway level, which involves the magnitude of pathway activity, spatial-temporal regulation, and non-canonical functions of the molecular switches in this pathway. This review discusses recent updates on the molecular mechanisms underlying the pathway-mediated growth inhibitory signaling, with a major focus on the regulation mediated at the pathway level.

## 1. Introduction

**Figure 1:**
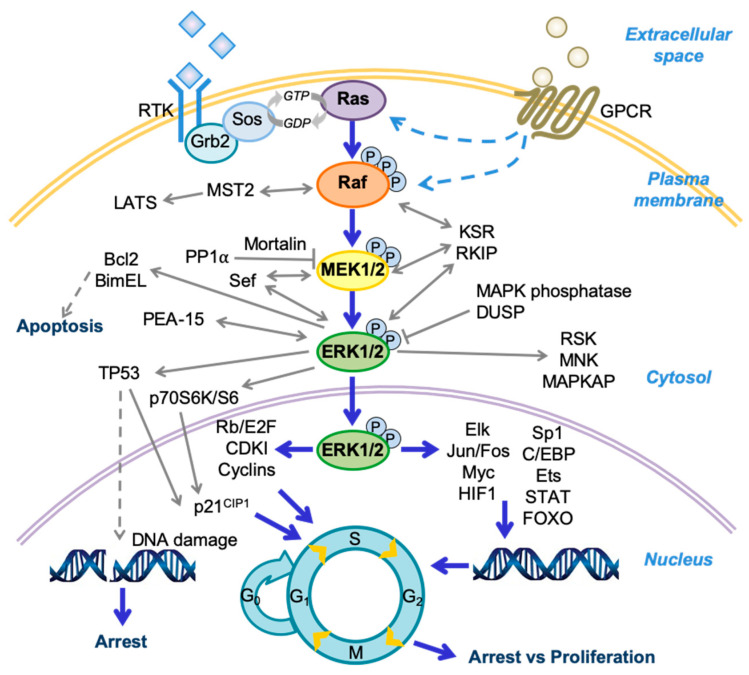
A schema of the Raf/MEK/extracellular signal-regulated kinase (ERK) pathway and its regulators and effectors. Growth factor receptor-bound protein 2 (Grb2); son of sevenless (Sos); large tumor suppressors (LATS); dual-specificity phosphatase (DUSP); ribosomal S6 kinase (RSK); mitogen activated protein kinase (MAPK)-activated protein kinase (MAPKAP); cyclin-dependent kinase inhibitor (CDKI); Hypoxia-inducible factor 1 (HIF1); CCAAT-enhancer-binding protein (C/EBP); signal transducer and activator of transcription protein (STAT); forkhead box protein O (FOXO); protein phosphorylation (P).

The mitogen activated protein kinase (MAPK) pathways allow cells to respond to various specific extracellular stimuli. There are four major and distinct MAPK cascades: extracellular signal-regulated kinase 1 and 2 (ERK1/2); c-Jun N-terminal kinase (1, 2, and 3); p38 MAPK (α, β, γ, and δ); and ERK5. The MAPK signaling participates in various biological contexts, ranging from early development to human diseases, with significant implications for cancer. As depicted in Figure 1, the Raf/MEK/ERK pathway is mainly activated through ligand stimulation of a receptor tyrosine kinase (RTK) on the plasma membrane, although this pathway can also be activated by a G-protein-coupled receptor (GPCR) via as yet unclear mechanisms (reviewed in [1,2]). The RTK signals are then transmitted by growth factor receptor-bound protein 2 (Grb2) and son of sevenless (Sos) to activate the small guanosine triphosphatase (GTPase) Ras, recruit Ras and the Ser/Thr kinase Raf (i.e., A-Raf, B-Raf, or C-Raf/Raf-1) to the plasma membrane followed by complex formation, and then activate Raf by inducing phosphorylation/dephosphorylation of several serine residues on Raf. Active Raf in turn phosphorylates and activates the dual-specificity kinases MEK1 and its homologue MEK2 (MEK1/2) at Ser218/Ser222 of MEK1 and Ser222/Ser226 of MEK2. MEK1/2 then sequentially phosphorylate Tyr and Thr residues in the TEY site (Thr202/Tyr204 of ERK1; Thr185/Tyr187 of ERK2) of the activation loop of their only known physiological substrates, the serine/threonine kinases ERK1/2. ERK1/2 then activate or inactivate a variety of proteins via phosphorylation in different subcellular compartments. While a number of ERK1/2 substrates have been identified to date (reviewed in [3,4]), their catalogue is likely to expand further due to continuing efforts in phosphoproteomics approaches that search for candidate ERK1/2 substrates (reviewed in [5,6]). The Raf/MEK/ERK pathway is regulated by a complex network of regulators, including additional small GTPases, phosphatases, scaffolds, and other kinases, which affects the magnitude, duration, and subcellular compartmentalization of the pathway activity. For more general information on molecular activity and regulation of the Raf/MEK/ERK pathway, readers are directed to review articles elsewhere [7,8,9,10].

Other than mediating diverse cellular processes, the Raf/MEK/ERK pathway is mainly known for its ability to promote cellular proliferation and survival and its deregulated activity is a hallmark of many epithelial cancers, wherein highly selective small molecule inhibitors of B-Raf and MEK1/2 are currently used for therapy [11,12,13,14]. Contrary to this, a significant body of evidence suggests that the Raf/MEK/ERK pathway can also mediate growth inhibitory signaling (reviewed in [15,16,17,18]). In addition to these in vitro evidences, growing evidence obtained from animal models and patient tumor tissue specimens for different tumor types suggests that this growth inhibitory signaling may also occur in vivo [19,20,21,22,23,24,25]. While this ability of Raf/MEK/ERK has significance in different biological contexts, including early development and neuronal differentiation, its significance in cancer is also noteworthy. For example, oncogenic mutants of RTK, Ras, or Raf can paradoxically induce growth inhibition in normal cells as well as in certain tumor cells, suggesting that this oncogene-induced growth inhibitory signaling may be an obstacle in the path of carcinogenesis [26,27,28]. Various mechanisms have been shown for their involvement in these phenomena, mainly including the cell cycle machinery that consist of Rb/E2F and cyclin-dependent kinase inhibitors p16^INK4A^ and p21^CIP1^, the tumor suppressor TP53 pathways, and different transcription factors and cell death machinery [15,16,17,29]. Indeed, genetic alterations, such as loss- or gain-of-function mutations and gene deletion that affect these mechanisms, are frequently detected in tumors and address how tumor cells bypass the control of growth. In addition, at the pathway level, spatio-temporal control can determine these paradoxical effects, as reviewed in [7,8,9,10]. Whilst these mechanisms have been addressed at a variety of downstream effector levels, our understanding of the mechanisms mediated at the Raf/MEK/ERK levels is relatively limited. In this review, we will discuss recent updates in the molecular mechanisms underlying growth inhibitory signaling of the Raf/MEK/ERK pathway with a major focus on its molecular switches and regulators.

## 2. Mechanisms of Raf/MEK/ERK Growth Inhibitory Signaling

### 2.1. Non-Canonical Effects of Raf Are Also Involved in Growth Inhibitory Signaling

A common hallmark of the growth inhibitory signaling mediated by the Raf/MEK/ERK pathway is the sustained activation of ERK1/2, which contrasts with the transient nature of most other ERK1/2-mediated cellular processes. As demonstrated by their constitutively active oncogenic mutants or chimeras of kinase domains, Raf proteins can sufficiently induce growth inhibition through their kinase function, which activates the MEK/ERK cascade (reviewed in [16,18]). Intriguingly, recent evidences suggest that non-catalytic Raf functions are also implicated in cell proliferative and growth inhibitory signaling. For example, a kinase-inactivating mutation (D594A) in B-Raf was shown to cooperate with oncogenic K-Ras to drive tumor progression in a C-Raf-dependent manner, as demonstrated in in vivo lung adenocarcinoma models [30,31,32]. Mechanistically, kinase-dead B-Raf hetero-dimerized with catalytically competent C-Raf and promoted K-Ras-mediated C-Raf activation [30,33]. Intriguingly, kinase-dead B-Raf was also shown to promote DNA damage, senescence, and apoptotic cell death at an early stage of KRAS tumorigenesis in an animal model [32]. Recent studies have revealed a co-regulatory mechanism involving C-Raf and A-Raf, wherein these Raf isoforms physically bind to, sequester and inhibit the pro-apoptotic kinase mammalian sterile 20-like kinase (MST2) independent of their kinase activity [34,35]. Intriguingly, A-Raf inactivated MST2 in the mitochondria in a scaffold protein kinase suppressor of Ras 2 (KSR2)-dependent manner by actively proliferating squamous epithelia and tumor cells [35]. Consistent with this, KSR2 depletion led to A-Raf dissociation from MST2 and re-location to the plasma membrane in non-malignant and differentiated squamous epithelia that underwent MST2-mediated apoptosis [35]. As such, C-Raf and A-Raf can affect cell proliferation and survival by regulating not only MEK/ERK but also MST-large tumor suppressor (LATS) pathways. Additional evidence supports kinase activity-independent effects of A-Raf in growth inhibitory signaling. For example, a kinase domain-deficient A-Raf splicing variant (DA-Raf) binds to Ras, suppresses MEK/ERK activities, and induces cell cycle arrest and myocyte differentiation [36]. This truncated A-Raf is unable to regulate MST2, suppresses K-Ras-induced transformation, and inhibits the proliferation of the human colon, head and neck, and lung cancer cells [37,38]. These studies demonstrate that all three Raf proteins have kinase-independent functions in addition to their canonical function as Ser/Thr kinases, indicating the complexity of pathway signaling at Raf levels.

### 2.2. Differential Regulation of MEK1 and MEK2 Levels in Cells

Constitutively active mutants of MEK1 and MEK2, generated by replacing the serine residues in activation loop with phosphomimetic aspartate (residues 218/222 for MEK1 and 222/226 for MEK2), are sufficient to phenocopy most, if not all, growth inhibitory effects of Raf in different cell lines (reviewed in [16,18]). It is known that signal amplification in the Raf/MEK/ERK pathway occurs more at the Raf-MEK step due to the greater molar ratio between Raf and MEK1/2 than between MEK1/2 and ERK1/2 [39]. Because MEK1/2 activate only ERK1/2 in most, if not all, biological contexts, whereas ERK1/2 serve as the focal points of the pathway signaling, thus no substantial contribution of MEK1/2 to signal amplification is expected, the importance of MEK1/2 in this ostensibly inefficient arrangement of molecular switches has been unclear. Nevertheless, it appears that cellular activity and expression levels of MEK1/2 are subject to dynamic feedback regulation. For example, we recently discovered that, under a condition wherein activated Raf induces p21^CIP1^ expression and subsequently growth arrest, MEK1 levels are transcriptionally upregulated, whereas MEK2 levels are downregulated due to decreased protein stability via ERK1/2-mediated feedback mechanisms [40]. In line with this differential regulation, knockdown of MEK1 blocked Raf-mediated p21^CIP1^ mRNA expression and growth arrest more effectively than MEK2 knockdown [40]. Of note, similar differential feedback regulation of MEK1 and MEK2 levels were also detected in a subset of B-Raf mutated tumor cells [40]. MEK1 and MEK2 are >86% identical at the amino acid level, and evaluation of their constitutively active mutants has revealed almost identical functional redundancy in various physiological contexts [41,42,43,44], although it was also noted that MEK1 and MEK2 exhibit distinct physical interactions with A-Raf [45] and the scaffold MEK Partner 1 (MP1) [46]. Further, many distinct effects reported in association with single depletion of MEK1 and MEK2 were mostly due to their differential expression, as demonstrated in different biological contexts, such as early development [47,48,49] and epidermal neoplasia [50]. As such, the differential effects of MEK1 and MEK2 knockdown on p21^CIP1^ expression and growth arrest are interpreted in a similar context. While the molecular mechanisms underlying these feedback MEK1/2 regulations require further elucidation, these findings add to the relatively short list of mechanisms regulating cellular MEK1/2 levels, which include Hu antigen R (HuR) regulation of MEK1 mRNA stability at its 3′ untranslated region [51].

### 2.3. Intrinsic Properties of ERK1/2 Affecting the Cell Fate Toward Growth Arrest Versus Death

ERK1 and ERK2, the bona fide substrates of MEK1/2, are highly homologous and functionally redundant. Although studies in mice have shown distinct effects of ERK1 and ERK2 ablation at different stages of development, including stem cell lineage commitment [52,53], T cell development [54], thymocyte maturation [55], and trophoblast development [49], studies also suggest that functional redundancy of ERK1 and ERK2 is evolutionarily conserved [56] and that differentially regulated expression of ERK1 and ERK2 mainly drive their biological differences [57]. ERK1 and ERK2 are also functionally redundant and interchangeable in Raf/MEK/ERK-mediated growth inhibitory signaling [43]. Intriguingly, we recently demonstrated that overexpression of ERK1 or ERK2 can switch C-Raf-induced growth arrest responses to caspase-dependent apoptotic death responses in different cell line models, which can then be reverted to growth arrest responses upon titrating the degree of ERK1/2 activation using MEK1/2 inhibitors [58]. Consistent with our observation, other groups showed that overexpression of ectopic ERK1/2 can induce robust cell death responses in a subset of human B-Raf^V600E^ melanoma cells [59,60]. Kinase function of ERK1/2 is crucial for these death effects, as catalytic site-disabled ERK2 mutants cannot induce cell death responses [58]. These phenomena suggest that the magnitude of ERK1/2 catalytic activity should be higher than a certain threshold to trigger cell death, while the availability of their death-specific substrates is also important in determining the cell fate. As such, it is possible that different cellular responses in the face of aberrant Raf/MEK activation, i.e., growth arrest vs. cell death observed in different cell types [16,18], might be partly attributed to different molecular composition in cells that affect the magnitude of ERK1/2 activity and the availability of context-dependent ERK1/2 targets. Of note, cellular ERK1/2 levels are subject to post-transcriptional mechanisms (reviewed in [61]), e.g., Pumilio2/PUM2 regulation of ERK2 translation [62]. It would be of interest to determine whether cellular ERK1/2 levels are correlated with cellular ability to display different growth inhibitory responses.

Of note, these lethal effects caused by ERK1/2 overexpression are quite contrasted with the effects of ERK1/2 mutants that contain a disabled active site but an intact activation loop. Although unable to mediate death responses, these ERK1/2 mutants could selectively restore growth arrest responses in Raf-activated but ERK1/2 knocked down cells, including p21^CIP1^ induction and E2F1 downregulation [43]. Therefore, it appears that different intrinsic properties of ERK1/2 in an active conformation are implicated in cellular growth inhibitory responses and that non-kinase ERK1/2 effects are involved in growth arrest signaling, while high magnitude ERK1/2 kinase activity is necessary for death signaling. Since the discovery of the effects of the diphosphorylated kinase-dead ERK2 mutant to activate topoisomerase II in vitro [63], kinase-independent effects of ERK1/2 have been reported in a few different biological contexts (reviewed in [64]).

### 2.4. Ectopic Expression of Autophosphorylating ERK2 Mutant Can Induce Cell Cycle Arrest

Given the strikingly high affinity between MEK1/2 and ERK1/2, relative to a typical enzyme-substrate interaction [65], ERK1/2 have been supposed as the focal point of Raf/MEK/ERK pathway signaling. Although the necessity of ERK1/2 for the pathway signaling has been demonstrated in various biological contexts, including cell proliferation [42,57,66], as well as growth arrest [43,44], demonstration of their sufficiency has been stymied, mainly due to the limit in deriving constitutively active ERK1/2 mutants because modification of their activation loop by phosphomimetic approaches do not make ERK1/2 active. As such, alternative approaches have been attempted, e.g., exploiting the synergistic mutations that facilitate autophosphorylation. Briefly, Tyr phosphorylation in the TEY site of the activation loop is critical for ERK1/2 to switch into active conformation, while subsequent Thr phosphorylation locks the kinase in the active conformation [67]. Therefore, phosphorylation of both residues is important to achieve maximal ERK1/2 activity in cells [67,68]. ERK1/2 can autophosphorylate their TEY sites [69,70,71]. Natali Ahn’s group demonstrated that Lys73Pro and Ser151Asp switches in rat ERK2 and facilitates the intramolecular interactions between Tyr on the TEY site and catalytic residues in the active site, which promotes ERK2 autophosphorylation and catalytic activity, albeit mildly [72,73]. Because the activation loop of this mutant, i.e., ERK2-L73P/S151D, is intact, its sufficiency for a physiological effect should be determined only in cells exhibiting low MEK1/2 activity. Our laboratory recently demonstrated that ectopic expression of ERK2-L73P/S151D mutant is indeed sufficient for inducing growth arrest in LNCaP cells and neurite differentiation in PC12 cells, which exhibit relatively low basal MEK1/2 activity [74]. Of note, this ERK2 mutant exhibits only mild kinase activity in cells and its overexpression did not induce cell death responses, which is in agreement with the notion that ERK1/2-mediated death signaling requires their high kinase activity.

The common docking (CD) site and F-recruitment site (FRS) are two major domains of ERK1/2 for physical interactions [10], which are independent of each other with respect to ERK1/2 catalysis [75]. Intriguingly, mutations that impair the CD site did not affect the growth arrest responses induced by ERK2-L73P/S151D or the aforementioned death responses induced by wild type ERK2 overexpression (Section 2.3) in Raf/MEK-activated cells [58,74]. In contrast, the FRS (Y261N) mutation markedly attenuated the death responses induced by wild-type ERK2 overexpression in Raf/MEK-activated cells [58], suggesting the significance of FRS in ERK1/2-mediated growth inhibitory signaling; the effects of Y261N could not be evaluated in ERK2-L73P/S151D because Y261N inhibited autophosphorylation of this mutant [74]. The F-site signature “Phe-Xaa-Phe-Pro” is relatively less frequent than the D-site signature and is found only in certain ERK1/2 substrates, including the cell-proliferative transcription factors ELK1, c-Fos, Fra1, and c-Myc, as well as the anti-apoptotic BH3-only protein BimEL [76,77,78]. Nonetheless, although ELK1 was required for ERK2-L73P/S151D to mediate PC12 differentiation and was not required for the ERK mutant to mediate growth arrest in LNCaP cells [74]. It is thus conceivable that a failure in relaying the ERK1/2 signal to these ERK effectors may partly contribute to the onset of growth inhibitory responses, while these responses would also be subject to cell type-specific expression of ERK1/2 effectors.

### 2.5. Regulators for Fine Tuning of Pathway Activity in Growth Inhibitory Signaling

Subcellular compartmentalization and temporal regulation of active ERK1/2 plays an important role in determining the physiological outputs of Raf/MEK/ERK signaling (reviewed in [9]) and are also important in growth inhibitory effects of pathway signaling (reviewed in [15]). These regulations are highly cell-type specific and mediated by a variety of proteins that directly interact with Raf, MEK1/2, and/or ERK1/2, which include scaffolds, anchors, and phosphatases [79,80]. As such, pathway access to an effector in a specific subcellular compartment or duration and magnitude of pathway activity can be precisely regulated. Multiple spatial and temporal regulators of the Raf/MEK/ERK pathway are known in the context of cell proliferative versus growth inhibitory signaling. A notable example is phosphoprotein-enriched-in-astrocytes (PEA-15), a 15 kDa acidic serine-phosphorylated protein that interacts with the common docking site of ERK1/2 via its C-terminal domain [81]. PEA-15 can promote ERK1/2 activation but sequesters ERK1/2 in the cytosol, limiting nuclear ERK1/2 activity and, consequently, ERK1/2-dependent cell proliferative transcription [82]. Consistent with this, ERK1/2 are mainly localized in the cytosol of cells undergoing Ras-induced senescence, mainly by virtue of PEA-15 [83]. Moreover, PEA-15-mediated sequestration of ERK1/2 in the cytosolic compartment can promote cell survival via autophagy [84], while PEA-15 depletion increases nuclear ERK1/2 in association with apoptosis in mouse testis cells [85]. Therefore, PEA-15 exerts anti-proliferative and anti-apoptotic effects via its ability to regulate ERK1/2 in a cell type-specific manner. Another example is similar-expression-to-FGF-genes (Sef), which can prevent ERK1/2 nuclear import while promoting cytoplasmic ERK1/2 activity by complexing with MEK1/2 and sequestering ERK1/2 in the cytosol [86,87,88].

The cell fate between proliferation and growth inhibition can also be determined by the magnitude of the signaling intensity, as demonstrated in a yeast model [89]. While PEA-15 and Sef regulate subcellular compartmental ERK1/2 activity, there are notable examples for the scaffolds that affect the magnitude of pathway activity. Kinase suppressor of Ras 1 (KSR1) is a well characterized molecular scaffold that promotes Raf/MEK/ERK activation and was previously shown for its requirement for Ras oncogene-induced senescence, as well as replicative senescence [90]. On the contrary, KSR1-mediated ERK1/2 activation was also necessary for cells to escape from cell cycle arrest induced by mitomycin C [91], suggesting that KSR1 regulation of ERK1/2 can induce different effects in opposing contexts. Another example is the Raf-kinase-inhibitor-protein (RKIP), which inhibits MEK1/2 phosphorylation by competitively interacting with C-Raf [92] and also suppresses MEK/ERK activity in BRAF-mutated melanoma cells [93]. MEK/ERK activity is also determined by the regulators that are located at the upstream tiers of the cascade, such as guanine nucleotide exchange factors (GEFs) and GTPase-activating proteins (GAPs) [8,94]. GEFs and GAPs can be expressed at different levels, which may affect the sensitivity of a cell to MEK/ERK. In support, loss of RasGAPs, via loss-of-function mutation or epigenetic inactivation, is common in cancers and correlates with poor prognosis in patients [95].

Inactivation of the pathway via dephosphorylation is also an important mechanism to regulate the pathway activity, and a number of phosphatases are known in this regard [80]. Nonetheless, the mechanisms underlying this negative regulation are less well known. We recently identified mortalin/HSPA9, a heat shock protein 70 paralog, as a negative regulator of Raf/MEK/ERK-mediated growth inhibitory signaling that functions at MEK1/2 levels via direct physical interaction [96]. We then demonstrated that mortalin can limit the pathway activity by promoting the physical interaction between MEK1/2 and the protein phosphatase PP1α via direct physical interactions through its C-terminal peptide binding domain, subsequently facilitating dephosphorylation of MEK1/2 [97]. These observations suggest that mortalin serves as a “rheostat”, which determines physiological output of Raf/MEK/ERK signaling. Intriguingly, although well known for its ability to sequester and inactivate the tumor suppressor TP53, mortalin depletion induced p21^CIP1^ transcription in TP53-deficient BRAF tumor cells, for which activation of the transcription factor Sp1 by upregulated Raf/MEK/ERK activity was necessary [98]. Hyperactivation of ERK1/2 can also lead to degradation of cell cycle regulators [99]. Currently, it is not known whether this mechanism is also involved in p21^CIP1^ regulation upon mortalin depletion in BRAF tumor cells. Because mortalin depletion or inhibition can induce lethality associated with altered mitochondrial permeability and bioenergetics in various tumor cells [100,101,102,103,104], it may be possible that mortalin has a role in coordinating oncogenic MEK/ERK activity and mitochondrial metabolism to facilitate tumor cell survival and proliferation.

## 3. Future Perspective

While a blockade of the activity of the Raf/MEK/ERK pathway is currently the mainstream strategy to treat RTK/Ras/Raf-driven tumors [11,12,13,14], many tumors exhibit innate or adaptive resistance to the therapies aimed at blocking the pathway activity. Although various mechanisms underlie therapy resistance, most of the resistance mechanisms converge at reactivation of MEK/ERK [105,106,107], indicating that this pathway remains as a key therapeutic target, even for therapy-resistant tumors. While various newer inhibitors are under development to continue the strategy of the pathway blockade, the ability of tumor cells to develop resistance foretells the limit of this strategy. Growth inhibitory signaling of the Raf/MEK/ERK pathway may indicate a potential for an additional strategy in targeting this pathway for tumor suppression, as supported by recent intriguing observations of tumor cell responses to hyper ERK1/2 activity. For example, discontinued drug treatment rendered regression of drug-resistant tumors in correlation with rebounded ERK1/2 activity in tumors cells [108]. Hyperactivation of ERK1/2, mediated by overexpression of oncogenic B-Raf or by depletion of dual-specificity phosphatase 6 (DUSP6), was also correlated with suppressed tumor cell growth in different cancer contexts [109,110]. In addition, ERK1/2 overexpression was sufficient to induce death of certain BRAF tumor cells [59,60]. A key question for future study is how to trigger this lethal potential of hyper ERK1/2 activity in tumor cells and whether a regulator of the Raf/MEK/ERK pathway can be exploited for that purpose. In this regard, close evaluation of known regulators of the pathway, as well as identification of additional regulators, ideally druggable, will be important.

## 4. Conclusion

The presence of growth inhibitory signaling of Raf/MEK/ERK suggests that cells must constrain the pathway activity within a desired range of signal intensity in order to achieve proper growth and proliferation. As such, not only too low but also too high signal intensity would be interpreted as a signal for cells to trigger anti-proliferative/survival responses. Further elucidation of the molecular mechanisms underlying this relatively less-known aspect of the Raf/MEK/ERK pathway signaling would broaden our understanding of this pathway and may offer an opportunity leading to the development of a novel therapeutic strategy.
